# A Split-Luciferase Reporter Recognizing GFP and mCherry Tags to Facilitate Studies of Protein–Protein Interactions

**DOI:** 10.3390/ijms18122681

**Published:** 2017-12-11

**Authors:** Mehdi Moustaqil, Akshay Bhumkar, Laura Gonzalez, Lisa Raoul, Dominic J. B. Hunter, Pascal Carrive, Emma Sierecki, Yann Gambin

**Affiliations:** 1 European Molecular Biology Laboratory Australia (EMBL Australia) Node in Single Molecule Science, Sydney NSW 2031, Australia; m.moustaqil@student.unsw.edu.au (M.M.); a.bhumkar@unsw.edu.au (A.B.); interacteambio@gmail.com (L.G.); d.hunter@imb.uq.edu.au (D.J.B.H.); 2 School of Medical Sciences, The University of New South Wales, Sydney NSW 2031, Australia; lisa.raoul@ens-rennes.fr (L.R.); p.carrive@unsw.edu.au (P.C.)

**Keywords:** protein–protein interaction, split-luciferase, universal reporter, *Leishmania tarentolae* cell-free

## Abstract

The use of fluorescently-tagged proteins in microscopy has become routine, and anti-GFP (Green fluorescent protein) affinity matrices are increasingly used in proteomics protocols. However, some protein–protein interactions assays, such as protein complementation assays (PCA), require recloning of each protein as a fusion with the different parts of the complementation system. Here we describe a generic system where the complementation is separated from the proteins and can be directly used with fluorescently-tagged proteins. By using nanobodies and performing tests in cell-free expression systems, we accelerated the development of multiple reporters, detecting heterodimers and homodimers or oligomers tagged with GFP or mCherry. We demonstrate that the system can detect interactions at a broad range of concentrations, from low nanomolar up to micromolar.

## 1. Introduction

It is currently estimated that over 650,000 protein–protein interactions exist in the human interactome [1] and constant efforts are ever-expanding this number. These interactions are central to cellular functions and are emerging targets for pharmacological intervention when implicated in a particular disease pathway [2]. Multiple in vitro and in vivo methods are reported in the literature to target and study these biomolecular interactions [3,4,5,6]. In vitro assays are mostly used for interrogating protein–protein and protein–DNA interactions and their antagonists. Commonly used techniques include variations of enzyme-linked immunosorbent assays (ELISAs) [7], surface plasmon resonance [8], and fluorescence polarization [9], which either require the use of antibodies or purified, often chemically derivatized, proteins. In vivo, powerful methods such as yeast two-hybrid assays [10] have the advantage of speed by eliminating the need for protein purification but can be subject to false positives and false negatives due to the multifactorial nature of signal generation [11]. Strategies to investigate protein–protein interactions (PPI) have become a crucial component in efforts to define the gene function, information flow, and organization of biochemical networks. It is with no surprise that in the last decade we have seen the emergence of new and optimized techniques to detect protein–protein interactions, both in vivo and in vitro.

Here we will focus more specifically on Protein Complementation Assays (PCA), also referred to as “split” systems [12]. In these assays, a reporter protein with enzymatic properties is “split” into two inactive fragments. Potential interacting proteins are fused separately with the two fragments. Upon interaction, the two fragments are brought into close proximity and re-assemble spontaneously into the active reporter. The first reconstitution of a functional protein from fragments was demonstrated in 1959 on a ribonuclease [13], but it was only in 1994 that the PCA was developed for PPI by Johnsson and Varshavsky [14]. They demonstrated that an ubiquitin (Ub)-based split-protein sensor could be used to examine the kinetics and equilibrium aspects of a PPI at its natural sites in a living cell. Since then, split-protein pairs have been developed using a variety of scaffolds from ubiquitin, GFP [15] and its variants [16,17], β-Galactosidase [18], dihydrofolate reductase (DHFR) [19], β-Lactamase [20], firefly [21], luciferase [22], or, more recently, thymidine kinase [23] and chorismate mutase [24]. These proteins support diverse enzymatic activities that can be varyingly difficult to quantify. The simplest assays are based on fluorescent proteins as detection of the fluorescent signal is straightforward [25,26]. On the other hand, luciferase is extensively used in biology to measure gene expression, as luminescence can be easily quantified on plate readers. Assays based on split-luciferases (split-luc) are therefore often chosen due to their ease of use and good sensitivity.

Even though PCA methods are extremely valuable tools for imaging and probing dynamic cellular processes, over the past decade, they have shown certain limitations. Long transfection times and the need to propagate cellular cultures prior to analysis are some of the main limitations in vivo. In vitro, it is mainly the extensive amount of protein purification required [27], associated with the proper folding of the recombinant proteins, that acts as a limiting factor. Last but not least, split-protein assays face other problems such as potential proteolysis of intracellularly-expressed proteins and peptides [28], as well as a lack of control over interfering co-expressed cellular factors.

However, the main limiting factor for PCA development is the need to design a specific reporter for each protein pair that is investigated. The PCA fragments need to be fused directly to the protein of interest, and the number of configurations to explore will rapidly increase the cloning costs [29,30]. Indeed, for each protein, one needs to choose which fragment to add, decide on the N-terminal or C-terminal tagging of the protein, and maybe try different linkers, and all these tasks are time-consuming and reduce the number of protein pairs studied. Here we discuss a new universal PPI and protein oligomerisation detector which can be adapted to any GFP- and/or mCherry-tagged protein system. Our system utilizes anti-GFP or anti-Cherry nanobodies fused to the split fragments of the newly-developed small luciferase nanoLuc (nLuc). In this study, we also take advantage of cell-free expression systems which help bypass the tedious protein purification steps allowing for rapid PPI detection [31]. This approach is already commonly used for rapid mapping of PPI [32], and here we have expanded it to address issues specific to a dynamic binary structure. We show that the new system can be successfully used to detect not only PPI and protein oligomerization, but can also be extrapolated as a competitor/inhibitor screen.

## 2. Results

### 2.1. Design and Universal Application

To design our reporter, we took advantage of nLuc, a small and bright luciferase which was systematically engineered from deep-sea luminous shrimp luciferase [33]. This was developed by Promega (Madison, WI, USA) into a protein complementation assay by splitting the full-length nLuc into a large bit (L, 18 kDa) and a small bit (S, 1.3 kDa).

**Figure 1:**
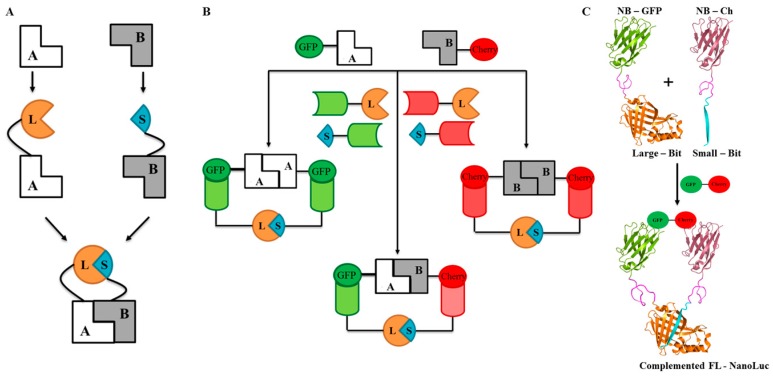
Schematic representations of protein complementation assays. (**A**): A generic split-luciferase assay is shown where the large bit (orange) (L—residue 1–162) and small bit (blue) (S—residue 163–174) of the split-luciferases (split-luc) (of which neither of them are active individually) are fused to interacting proteins A and B. Upon successful interaction between A and B, the L and S reconstitute resulting in luminescence [33]; (**B**): Schematic representation of a universal binary reporter system where the Large bit (L—orange) and small bit (S—blue) of the nLuc are fused with GFP-/mCherry-nanobody (light green and light red respectively) at either the N-/C-terminal. The fused proteins are separated by a flexible linker (GSSGGGGSGGGGSSG). This universal reporter system can detect either protein homodimers for GFP-tagged proteins (green) (eGFP-A:A-sGFP) and mCherry-tagged proteins (mCherry-B:B-mCherry), or heterodimers of GFP- and mCherry-tagged (red) proteins (eGFP-A:B-mCherry); (**C**): Crystal structure representation of the universal binary reporter split-luc assay of GFP-nanobody, split-luc large bit, and the small bit [33,34]. The anti-GFP nanobody (light green) is fused with large bit (orange) of nLuc and the anti-mCherry nanobody (light red) is fused with small bit (blue). This complex when mixed with GFP-mCherry heterodimer (green and red respectively), the binding of anti-GFP and anti-mCherry nanobody to their respective partners facilitates the complementation of full-length nLuc reporter.

The traditional split-luciferase assay is designed by fusing the individual bits of split-luciferase (L and S) to the interacting proteins (A and B) (Figure 1A). This involves several rounds of cloning using restriction enzyme digestion and then subsequent ligation which is specific to every protein pair that is investigated. To create a generic split-luciferase system which can be used with any GFP- and mCherry-tagged target proteins, we fused the large (L) and small (S) bits of nLuc to the N- or C-terminus of either the anti-GFP (G) or anti-Cherry (C) nanobody. A gateway site-directed recombination technique was used to create the fusion proteins and these were tagged with 8× His tag to facilitate purification. Our approach yields eight possible combinations of split-luc nanobody fusion peptide (SLN) which can be used to detect homo- and heterodimers (Figure 1B). These eight SLNs were labelled as GL, LG, CL, LC, GS, SG, CS, and SC, based on the terminal of the nanobody where the nLuc bit is fused. For example, GL is the construct that has an anti-GFP nanobody (G) followed by the large (L) bit of nLuc. SC corresponds to the construct where the small (S) bit of nLuc is followed by the anti-Cherry nanobody (C). The nanobodies and split nLuc bits are separated by a 15-amino acid flexible linker (Table S1), which allows rotational and directional flexibility for optimal recombination upon successful protein–protein interaction (Figure 1C).

The nanobodies have binding affinities in the sub-nanomolar range (0.49 nM for GFP and 1.4 nM for Cherry), outcompeting the intrinsic affinity of the nLuc subunits (190 µM), so we expect that the split-luciferase complementation will not be self-driven but will depend on PPI [33,34,35]. To validate this, every luciferase assay is carried out using cell-free lysate alone (Figure S1) as negative control. At all concentrations of SLNs, luminescence was at background level suggesting that self-recombination of the bits was negligible.

### 2.2. Proof of Principle and Calibration of the PPI Assay

To test the ability of the system to detect protein homo- and hetero-dimers, eGFP–sGFP, mCherry–sGFP, and mCherry–mCherry tandems were used as models and tested against all 8 potential pairs of SLNs in an 8 × 8 matrix. The targets and the SLNs were both expressed in *Leishmania* cell-free expression system, using *Leishmania tarentolae* extracts (LTE) and were 10-fold diluted post-expression before using them for the PCA. Each of the assay components were mixed in the ratio of 1:1:1 and incubated for 10 min at 23 °C before adding the nLuc substrate.

**Figure 2:**
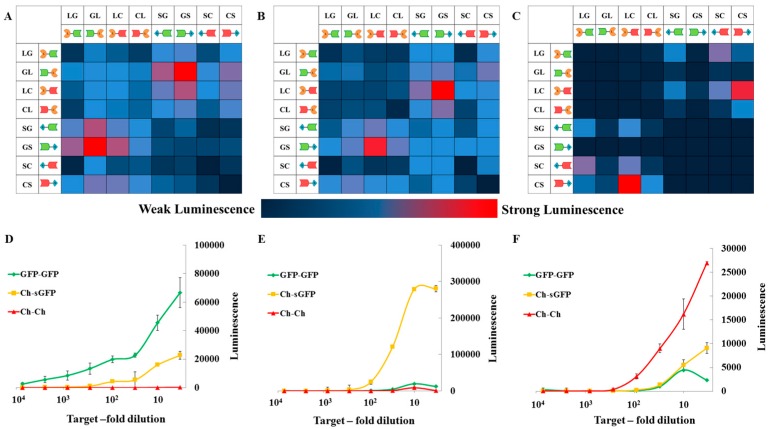
Heat-map and graphical representations of the selectivity of specific split-luc nanobody fusion peptide (SLN) bit pairs. (**A**–**C**): Heat-map representations of luminescence observed with 8 × 8 matrices of SLN bits as tested on eGFP–sGFP homodimer (**A**), mCherry–sGFP heterodimer (**B**), and mCherry–mCherry homodimer (**C**). The heat-map represents higher luminescence signals in red with weaker luminescence transiting towards the blue. As expected, SLNs self-binding give the least signal of all the targets; (**D**–**F**): Graphical representation of luminescence signals for individual targets plotted against the serial dilution of SLNs. The assay was run using the best SLN pairs as observed from the 8 × 8 matrix. The SLN pairs used for the target proteins were: GL–GS (**D**), LC–GS (**E**), and CL–CS (**F**). Each of the pairs were tested on all the targets and their signals are plotted in individual graphs as eGFP–sGFP (green), mCherry–sGFP (orange), and mCherry–mCherry (red). The average ± SEM for at least three experiments is plotted. LG: Large bit–anti-GFP, GL: anti-GFP–Large bit, LC: Large bit–anti-Cherry, CL: anti-Cherry–Large bit, SG: small bit–anti-GFP, GS: anti-GFP–Small bit, SC: Small bit–anti-Cherry, CS: anti-Cherry–Small bit.

In each case (eGFP–sGFP, eGFP–mCherry, or mCherry–mCherry), signals were detected with excellent signal-to-background ratios and with at least one configuration of the reporter. As shown in Figure 2, the best hits were GL/GS (Figure 2A, eGFP–sGFP homodimer), LC/GS (Figure 2B, mCherry–sGFP heterodimer), and CL/CS (Figure 2C, mCherry–mCherry homodimer). For all 8 × 8 matrices, the large–large and small–small self-associations of SLNs gave the lowest signal. The best hits from our 8 × 8 matrices were more extensively tested against all target proteins. To validate our data in all cases, we used other constructs as negative controls. For example, in the eGFP–sGFP detection assay, (Figure 2D) both mCherry–mCherry and mCherry–sGFP acted as background controls. At all dilutions of the SLN, each individual nLuc pair showed maximum reactivity to its target, with a signal-to-background ratio of at least 3, and at best, more than 30 (Figure 2D–F).

The resulting matrices also showed that a split-luciferase pair in one orientation can perform very much better (sometimes more than 10-fold) than in other conformations (e.g., in comparing GL/GS and LG/GS (Figure 2A)). Despite the fact that the reporters were designed to have a flexible linker separating the Luc bits from the nanobody, the sheer size of the nanobody and the interacting GFP- and/or mCherry-fused proteins could be a limiting factor. The reporter may be able to complement in only one orientation depending on the size of the interacting protein pair and its mode of binding. From this initial screen, two homodimer reporters (GL/GS and LC/CS for GFP- and mCherry-homodimers respectively) and one heterodimer reporter (LC/GS) were selected.

**Figure 3:**
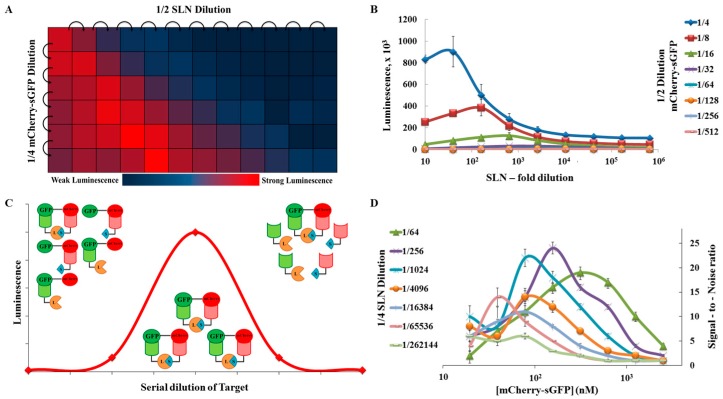
Detection limit studies using a direct approach. (**A**): Heat-map of the ‘hook’ effect; the ‘hook’ effect transition is seen when we plot the luminescence values of SLN and target dilution as a heat-map. Here the highest luminescence is plotted in red and weaker luminescence transits towards blue; (**B**): Graphical representation of the ‘hook’ effect as seen with the mCherry–sGFP tandem serial dilution of the LC–GS SLN pair. We see a shift in the ‘hook’ effect with increasing dilution as the 1:1 ratio of target to SLN shifts to the right; (**C**): Representation of the ‘hook’ effect as seen in the split-luc assay. With serial dilution of the target, we see less luminescence when the SLN concentration is less than the target or vice-versa. However, we reach the maxima when the proportion of the SLN and the target proteins reach a 1:1 ratio, giving the highest luminescence; (**D**): Graphical representation of the signal/noise ratio from the data obtained for graph 3B. The data was plotted by taking the ratio of the luminescence for the target protein and the luminescence observed for the lysate background at the same concentration of SLN. In this case too, we see a ‘hook’ effect that moves with changes in the concentrations of target and reporter.

One limitation of the cell-free expression system that we use is that the levels of expression can be low (in general 1–2 µM and sometimes 10-times lower, especially when proteins are coexpressed [36]). This feature can be beneficial as the concentrations are often at physiological levels, suggesting that the detected interactions could be relevant and specific. However, to detect these interactions and be useful *in cellulo*, the PPI-detection method has to be highly sensitive. From our initial screen, we knew that our system was working in the cell-free extracts. Thus, we wanted to see how the system behaves at different target and SLN concentrations. This was established for the detection of mCherry–sGFP tandems by the LC/GS reporter. Serial dilutions of the target (mCherry–sGFP) and the reporter (LC/GC) were used corresponding to concentration ranges from 5 to 250 nM and around 0.2 to 1000 nM, respectively. At the “normal” concentrations of SLN (estimated at 1 µM), the signal decreased as mCherry-sGFP was diluted. Compared to a “lysate only” control, the presence of mCherry–GFP could be satisfyingly detected above 10 nM (Figure 3A). However, when both the SNLs and the target concentrations were modified, the luminescence maxima shifted with the SNL dilution (Figure 3A,B). A significant signal could still be detected with as low as 10^5^-fold dilution of SNL (Figure 3D). This can be rationalized: the signal follows a “hook” pattern wherein maximum signal is detected when the SLN bits and the target proteins are in a 1:1 ratio (Figure 3C). At high concentrations of target proteins, the two parts of the split system may distribute on different tandems and are unable to recombine. With the dilution of the targets, as the target-to-SLN ratio reaches 1:1, it generates maximum signal, as seen in our assays. With further dilutions, the reduction in the number of targets results in loss of signal. It means that, by diluting the SLNs (i.e., reporters), we can shift the optimal range of detection for the interacting protein pair using self-calibration of the system. This means that we can still detect sub-nanomolar concentrations of the target (Figure 3D). Based on the protein concentration, our assay can detect interacting protein pairs (mCherry–sGFP in this case) at 1 nM, diluting the SLN in the high pico-molar range with a signal-to-background ratio greater than 5 (Figure 3D). Therefore, the assay should be able to work to detect PPI even with low protein expression.

**Figure 4:**
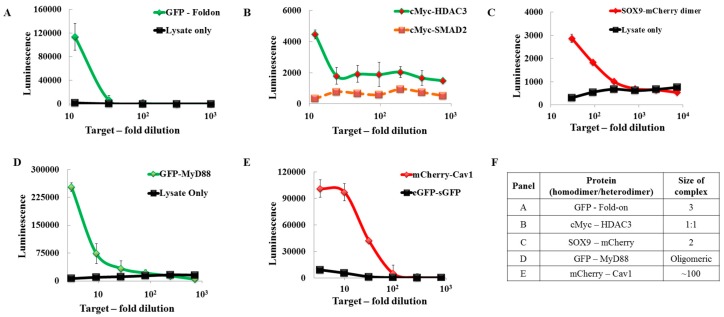
Applying the split-luc reporter to PPI and self-dimerising/oligomerising proteins. (**A**): Graphical representation of split-luc assays performed on the sGFP–Foldon protein. sGFP–Foldon is a trimer wherein GFP trimerizes post-expression due to the foldon domain. We could detect a high luminescence signal over the background for sGFP–Foldon using the GL–GS SLN pair; (**B**): Graphical representation of split-luc assays performed on the cMyc (b/HLH/Zip)–HDAC3 heterodimer. For this assay, cMyc and HDAC3 were co-expressed in *Leishmania* cell-free lysate and their serial dilutions were used in the split-luc assay using the LC–GS SLN pair. As a negative control, the non-interacting protein pair cMyc–SMAD2 (dotted line) was used; (**C**): Graphical representation of split-luc assays performed on the SOX9–mCherry homodimer. The SLN pair of CL–CS was used for this assay with a lysate-only control used to detect background luminescence; (**D**): Graphical representation of split-luc assays performed on MyD88 using the GL–GS SLN pair; (**E**): Graphical representation of split-luc assay performed on mCherry–Cav1 using the LC–CFRS SLN pair. As a measure of background luminescence, the eGFP–sGFP homodimer was used; (**F**): Expected stoichiometry of each target [32,37,38,41,42]. The accession numbers of all the genes used are mentioned in Table S2.

All the previous tests were performed using fused peptides. To evaluate how the system performed on “real” proteins pairs or oligomers, known PPI were selected for each reporter: eGFP–foldon [37] (homotrimer of GFP), sGFP–MyD88 [38], the cMyc(b/HLH/Zip)–HDAC3 [39] pair (heterodimer of sGFP-mCherry), mCherry–SOX9 [40] (mCherry homodimer), and mCherry–Cav1 [32] (Figure 4). For detecting these interacting proteins, the targets and SLNs were separately expressed in *Leishmania* cell-free lysate and the targets were serially diluted three-fold before adding them to the assay (detailed experiment outlined in the material and methods section). To test the background luminescence originating from the SLN self-interaction, non-expressing *Leishmania* cell-free lysate was used and it too was serially diluted three-fold before adding it to the assay. In every case, we were able to detect a luminescence signal with a signal-to-background ratio of at least 5 (mCherry–SOX9) and at best 60 (sGFP–MyD88). To establish a non-interacting pair as a negative control, along with cMyc–HDAC3, cMyc–SMAD2 was used. The luminescence from the cMyc–SMAD2 PPI pair was as low as background levels typically measured for a non-expressing LTE control.

### 2.3. Application to Measure PPI Disruption

**Figure 5:**
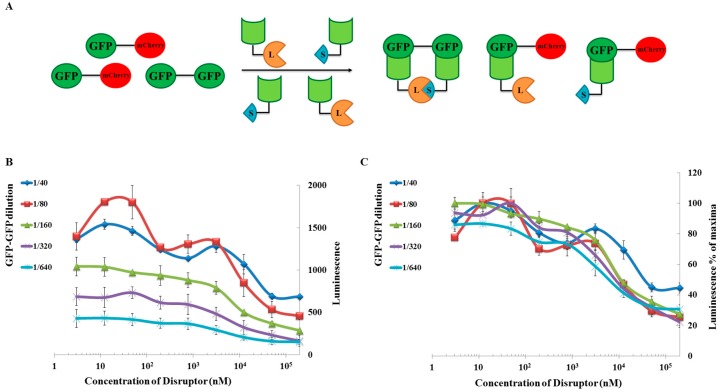
Detection limit studies using a competition approach. (**A**): Schematic representation of the indirect approach that was used to study the detection limit of the split-luc assay. For the assay, along with the SLN bits and the target protein, we expressed the competitor in a *Leishmania* cell-free system, and this was used for our assays. The competitor was incubated with SLN bits allowing it bind to the nanobody before adding the target protein and the substrate; (**B**): Graphical representation of luminescence as a function of competitor concentration; (**C**): Normalized data-set from the data obtained for graph 5B. The data was normalized against the maximum luminescence obtained for each target concentration and plotted against the competitor concentration. The data-set for each target concentration seems to overlap, suggesting that the inhibition curves obtained were independent of target protein concentration.

Along with standard PPI detection, we wanted to test if the split-luc system could be modified to screen for PPI inhibitors. First however, we determined the characteristics of the assay by creating a competition between eGFP–sGFP (target) and mCherry–sGFP (competitor). This is not an inhibition assay but rather, it mimics a “cold” probe assay (Figure 5A). The mCherry–sGFP competitor, SLNs, and eGFP–sGFP target were expressed in *Leishmania* cell-free lysate. A single SLN concentration was chosen for this study which would give us a detectable range of signal-to-background ratio while not being a rate-limiting factor (in practice 20-fold dilution for an estimated concentration of 50 nM was used). Post-dilution, the SLNs were pre-incubated for 10 min at 23 °C with varying concentrations of mCherry–sGFP before adding the desired concentration of the eGFP–sGFP target to the assay. For all eGFP–sGFP concentrations tested, we saw a decrease in luminescence with increasing concentrations of the mCherry–sGFP tandem. The decrease in luminescence could be detected for competitor (mCherry-sGFP) concentrations of as low as 6 nM (Figure 5B). In our case, the assay responded correctly for target (eGFP–sGFP) concentrations between 0.8 and 12 nM (i.e., 1/640 dilution and 1/40 dilution). In this range, the inhibition curves, when normalized against their respective maxima, showed a perfect overlay, suggesting that the mCherry–sGFP inhibition was independent of eGFP–sGFP concentration (Figure 5C) as it should be since we are testing the binding of the anti-GFP nanobody to its substrate. Importantly, this means that in the case of direct detection described above, the competition assay can be performed at different concentrations of target without affecting the results.

**Figure 6:**
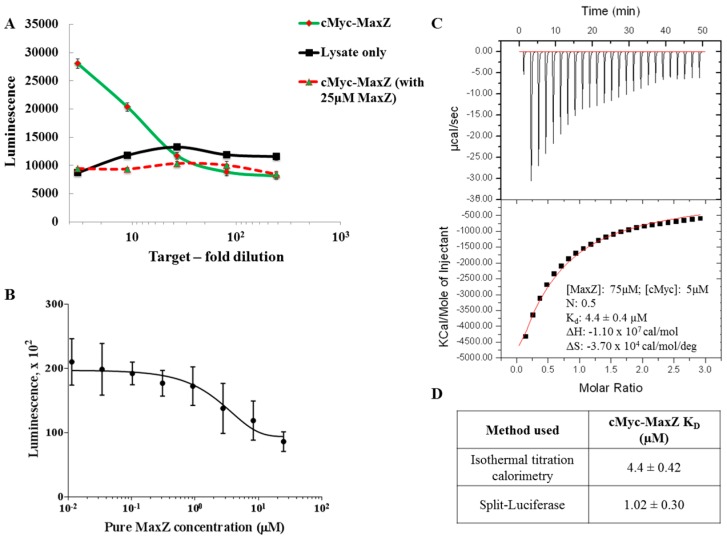
Using split-luciferase assay to determine apparent *K_D_*: (**A**): Graphical representation of split-luc assays performed on the cMyc–MaxZ PPI pair. Here, the non-expressing *Leishmania tarentolae* extract, (LTE) acts as a negative control (in black). To test if the PPI can be disrupted by pure MaxZ, we used 25 µM MaxZ in conjugation with the PPI pair in the split-luc assay. Here, we do see a reduction in luminescence to the background level, due to presence of pure MaxZ; (**B**): Binding studies of cMyc–MaxZ using purified MaxZ as a PPI disruptor; (**C**): iTC titration curve for cMyc–MaxZ titration where 75 µM MaxZ was titrated in 5 µM cMyc and ‘one set of site’ fitting was used to fit the data to get a *K_D_* of 4.4 µM; (**D**): Table comparing the binding constants for cMyc–MaxZ using iTC and split-Luc assay.

Competition between proteins for binding can be used to evaluate the apparent *K_D_* of a protein pair. Here we used it to measure the well-described interaction between cMyc (b/HLH/Zip) and MaxZ (b/HLH/Zip) [41]. First, we verified that the protein pair could be detected by our assay. Indeed, when cMyc–mCherry and MaxZ–sGFP were co-expressed in LTE and then added to the LC/GS pair of SLNs, a luminescence signal could be detected well above the background level obtained with lysate only (Figure 6A, black line). When 25 µM of purified MaxZ was added to the assay, a reduction of the luminescence signal was observed (Figure 6A, dotted line). Titration of purified MaxZ into the assay yielded a relationship between luminescence and concentration that could be fitted to reveal the apparent *K_D_* of the interaction (*K_D_* = 1.02 µM, *r*^2^ = 0.8%) (Figure 6B). This value is in good agreement with the reported literature where cMyc–MaxZ *K_D_* is reported to be in the range of 0.3–76 µM and also corresponds to the *K_D_* value (*K_D_* = 3.2 µM) we obtained using Isothermal Titration Calorimery (ITC) (Figure 6C) [43,44]. Therefore, our generic assay can be adapted to measure affinity constants of protein pairs.

## 3. Discussion

Genetically-encoded fluorophores are now commonly used in fluorescence microscopy but can also act as purification tags [43,44,45], folding/solubility markers [46,47], or be developed into functional assays [48,49,50]. To be able to study PPI and protein oligomerisation using constructs already in use, we created a generic luminescent PCA reporter that can recognize GFP- or mCherry-tagged proteins. Our in-house developed *Leishmania* cell-free expression system allows us to quickly assess the performances of the assay in multiple configurations. The strong affinity of the GFP and mCherry nanobodies helps push detection limits to lower than has been reported [33]. Owing to its design, by changing the concentration of the reporter, this assay can be applied to an extensive range of target concentrations, from a few micromoles down to nanomolar range, without losing efficacy. This implies that the same assay can be used with similar efficacy on proteins from different sources (from concentrated purified recombinant proteins to proteins in cell extracts at physiological levels) without the need to extensively modify the procedure, and can probably be readily implemented using constructs developed for other uses. Even though the assay should work with higher concentrations of proteins, it is always better to use a more dilute preparation in order to avoid unspecific aggregation of the proteins themselves. The monomeric GFP and Cherry tags were designed to minimize self-association compared to other fluorescent proteins that could form tetrameric assemblies. Our previous experiments testing GFP- and mCherry-tagged proteins using single molecule fluorescence detection support our claim that we do not see any endogenous oligomerization associated with fluorescence tags [32,36,38,42,51]. Still, as the split-nLuc assay is very sensitive, the self-association of the fluorescent tags could create false-positive signals and would need to be taken into account at high protein concentrations. Diluting the targets also means that the SLNs can be used at a lower concentration, preventing untriggered self-association and decreasing background fluorescence.

From a pharmaceutical point of view, this can be very valuable. PPI are becoming a very important class of drug targets. When crucial PPI become deregulated, cellular processes are altered; screening for regulated PPI and understanding their role in health and disease [52] is therefore attractive. PPI detection and PPI disruption assays are important in almost all steps of drug discovery, including target identification, assay development, hit identification-validation, lead optimization, and preclinical validation. For example, studying the interactome of a key cellular protein can lead to target identification, disruption of specific PPI in vitro, and will provide the lead compound. On the other hand, in vivo validation of drug effects can require monitoring the loss of an interaction in an organism. Our generic assay could evolve to be very valuable in this perspective as it is able to test different protein pairs without rounds of cloning; it can also be used from cell extracts or purified proteins. It could allow for sensitive and kinetically robust measurements of PPI, and with further developments, be adapted to high throughput applications. So far, we are expressing the SNLs in LTE for each experiment but this is a source of variability. Using purified recombinant SNLs should not only reduce day-to-day variability, but we also expect it to further reduce the background fluorescence, ultimately increasing the signal-to-background ratio of the assay.

The most valuable aspect of our assay is its ability to efficiently detect protein oligomers and aggregates. Protein aggregation is now recognized as a defining component of numerous human disease states including, Alzheimer’s (Aβ protein), Huntington’s (huntingtin protein), and Parkinson’s (α-synuclein protein) [53]. A generic reporter should be able to detect both PPI and self-oligomers. Here, by changing the SLN pair used for the assay, we can adapt the assay to detect PPI (LC/GS) or GFP- or mCherry-tagged protein aggregates (GL/GS and CL/CS) (Figure 4). The signal generated by the presence of oligomers/small aggregates is often larger than the one corresponding to a simple PPI (compare A and B, Figure 4) and can serve as a quick “diagnostic” of protein aggregation. Importantly, when using the PPI reporter (Cherry/GFP recognized by the LC/GS pair respectively), one will only focus on the PPI and ignore the individual GFP/ Cherry oligomers. The parallel use of the three reporters in the same assay format gives a complete picture of the dynamics of the interaction. The self-assembly reporter can provide a simple assay for identifying compounds capable of modulating the aggregation process.

## 4. Materials and Methods

### 4.1. Gateway Cloning and Protein Expression

The gene sequences for the large and the small bits of the split-luc system were obtained from the Promega Nano-Glo^®^ Luciferase Assay System (Madison, WI, USA). ORFs were generated by adding the GFP/mCherry nanobody sequences to either N-/C-terminal of the luc bits, with a 15-amino acid flexible GS linker separating the nanobody and the luc bit. These ORFs were ordered as G-Blocks from IDT and were cloned into the Gateway pDONR221 vector using Gateway™ BP Clonase™ II from ThermoFisher Scientific (Waltham, MA, USA). Subsequently, the resulting entry vectors were recombined with the cell-free expression-specific Gateway™ destination vectors (pCellFree5795) using Gateway™ LR Clonase™ II from ThermoFisher Scientific. The destination vectors chosen did not have any intrinsic fluorophore, but had an N-terminal 8× HIS tag to facilitate purification. The resulting clones were then transformed, colony picked from α-select competent cells, and the plasmid DNA was extracted using ZymoPURE™ Plasmid Midiprep Kit (Irvine, CA, USA). For the cell-free expression of these proteins, the plasmid DNA with the ORFs was added to *Leishmania tarentolae* cell-free lysate (supplemented with feed solution and Mg^2+^) in a ratio of 1:9 [32,51,54,55]. The protein was allowed to express for two and half hours at 27 °C, before using it for downstream processing.

### 4.2. Split-Luciferase Assay for Direct Detection Studies

The SLNs and the target protein were expressed individually in LTE by adding 1 µL of plasmid DNA to 9 µL of LTE. The expressed SLNs or the target proteins were serially diluted in buffer A (25 mM HEPES pH 7.4, 50 mM NaCl), depending on the assay. For the direct binding assay, each SLN from a large and small bit pair was mixed with target protein in a 1:1:1 ratio (2 μL:2 μL:2 μL). This mixture was incubated for 10 min at 23 °C and 6 µL of luciferase substrate (after diluting it as per the manufacturer’s recommendation) was added to the mix. The final mixture of SLNs, target protein, and luciferase substrate was incubated for another 10 min at 23 °C before reading the luminescence on a PerkinElmer EnSpire plate reader (PerkinElmer, Waltham, MA, USA), using the manufacturer’s recommended settings for luminescence assays (0.1 s read per well).

### 4.3. Split-Luciferase Assay for Indirect Detection Studies

SLNs and target proteins (eGFP–sGFP) were expressed individually in LTE by adding 1 µL of plasmid DNA to 9 µL of LTE. The competitor (mCherry–sGFP) was also expressed in LTE and it was serially diluted in buffer A and mixed in a 1:1:1 ratio with the SLN pair. This mixture was incubated for 20 min at 23 °C before adding 2 µL of target protein to the mix. To initiate the luciferase reaction, 6 µL of luciferase substrate (after diluting it as per the manufacturer’s recommendations) was added to the mix and luminescence was read on a PerkinElmer EnSpire plate reader after 10 min of incubation at 23 °C.

### 4.4. Split-Luciferase Assay for Apparent K_D_ Calculation

To detect the apparent *K_D_* of the cMyc–MaxZ interaction, MaxZ was purified as per mentioned in Park et al. [56]. For the split-luc assay, MaxZ–sGFP, cMyc–mCherry, and the SLNs (LC and GS) were expressed in LTE (as mentioned above). For the protein pair, cMyc and MaxZ single expressions were mixed in a 2:1 ratio after 10 min of expression and the mixture was expressed further for 2 h at 23 °C before using it for the assay. The SLNs were serially diluted three-fold and were mixed at different concentrations (serial dilutions from 25 to 10 nM) of purified MaxZ. This mixture was incubated for 20 min at 23 °C before adding the protein pairs MaxZ–sGFP and cMyc–mCherry in equal ratios. Finally, luciferase substrate was added to initiate the reaction and luminescence was read on a plate reader after 10 min incubation at 23 °C. To calculate the *K_D_* of the binding, the curves were fit using the one-phase exponential decay equation (Y = (Y0 − Plateau)·exp (−*K_D_*·X) + Plateau) in GraphPad^®^ Prism.

For measurement of cMyc–MaxZ *K_D_* using iTC, both the proteins were purified as per mentioned in Park. et al. [56]. These proteins were dialyzed in Buffer A using GE Healthcare Sephadex G-25 in PD-10 Desalting Columns and were further concentrated to the desired concentration using Merck Amicon^®^ (Kenilworth, NJ, USA) 10 kDa cut-off concentrators. GE Healthcare iTC200 (GE Healthcare, Chicago, IL, USA) was used for our studies wherein 75 µM MaxZ was titrated in 5 µM cMyc using multi-injection mode (20 injections of 2 µL each). The data was then analyzed using Origin^®^ software (Version 5.0, GE Healthcare, Chicago, IL, USA) and the curve was fit for the “one set of sites” equation giving the cMyc–MaxZ *K_D_* of 4.4 µM [57].

### 4.5. Split Luciferase Assay for PPI, Homodimerisation, and Oligomerisation Detection

To study the PPI using split-luc assays, the interacting pairs of GFP- and mCherry-tagged proteins were individually expressed in LTE for 10 min (as per mentioned above). These protein pairs were subsequently co-expressed by mixing GFP-expressing LTE with mCherry-expressing LTE in a ratio of 1:2 and the mixture was incubated for another 2 h at 27 °C. In case of homodimerising and oligomerising proteins, the individual GFP-/mCherry-tagged proteins were expressed in LTE (as mentioned above) before being used for the split-luc assay. To facilitate better detection and get higher signal-to-background ratios, we developed a longer linker to study mCherry-tagged proteins. This linker was made by combining flexible and rigid linkers (Table S1). This linker was used in conjugation with anti-mCherry nanobodies and small bits from nLuc to form the CFRS SLN (anti-mCherry–Flexible–rigid linker–small bit). The SLN pair LC/CFRS was then used as an mCherry-oligomer detector. The PPI pairs, homodimers, and oligomers were treated like target protein in the split-luc assay and the resulting luminescence was read on a plate reader after 10 min incubation at 23 °C.

To detect the background luminescence, the non-expressing LTE serial dilution or single dilution was mixed with an equal volume of SLN pairs. This mixture was subject to the same treatment as the target protein and the resulting luminescence was read on a plate reader.

## 5. Conclusions

In conclusion, our generic split-luciferase reporter is designed for the rapid investigation of PPI as well as low limit detection of protein aggregation. Benefiting from the small size and bright luminescence of nLuc, it allows for detection at low concentrations with minimal interference coming from self-association. Notably, conjugating this system with a *Leishmania* cell-free expression system sidesteps many of the limitations of comparable PCA routinely used in protein analysis. Our assay is distinct from similar PCAs as it can be adapted to any GFP-/mCherry-tagged system and optimized for high throughput screening. Our analysis indicates that this method fulfils the general expectations of a complementation reporter in vitro, with biomolecular parameters generally suited for accurate detection of PPI. In addition to this, in detecting lower levels of protein aggregation, we open up the ability to generate potential pharmacological outcomes. Specifically, this assay can reveal drug potency for induced protein interactions by disrupting the luminescence signal and can also measure the efficiency of drugs (IC_50_). Moreover, with the aptitude of this generic binary reporter, we believe that this luminescence system will provide additional applications yet to be realized. A faster and better understanding of the underlying mechanism of cellular signaling, protein interaction networks, and drug interactions will help improve therapeutic interventions.

## Supplementary Materials

Supplementary materials can be found at www.mdpi.com/1422-0067/18/12/2681/s1.

## Abbreviations

PPI	Protein–Protein Interaction
sGFP	superfolder GFP
eGFP	enhanced GFP
mCherry	monomeric Cherry
PCA	Protein Complementation Assays
DHFR	Dihydrofolate Reductase
nLuc	nanoLuc luciferase
L	large bit of the nanoLuc 18kDa
S	small bit of the nanoLuc 1.3kDa
G	anti-GFP nanobody
C	anti-Cherry nanobody
SLN	Split Luc Nanobody fusion peptide
GL	anti-GFP–Large bit
LG	Large bit–anti-GFP
CL	anti-Cherry–Large bit
LC	Large bit–anti-Cherry
GS	anti-GFP–Small bit
SG	small bit–anti-GFP
CS	anti-Cherry–Small bit
SC	Small bit–anti-Cherry
CFRS	anti-Cherry flexible-rigid linker small bit
LTE	*Leishmania tarentolae* Extract
