# Supplementary material for: A Split-Luciferase Reporter Recognizing GFP and mCherry Tags to Facilitate Studies of Protein–Protein Interactions
Source: Int J Mol Sci. 2017 Dec 11;18(12):2681. doi: 10.3390/ijms18122681 (PMC5751283; doi:10.3390/ijms18122681)
Supplement: Supplementary file 1 [file ijms-18-02681-s001.pdf]

International Journal of *Molecular Sciences*

MDPI

A split-luciferase reporter recognizing GFP and mCherry tags to facilitate studies of protein-protein interactions

Mehdi Moustaqil, Akshay Bhumkar, Laura Gonzalez, Lisa Raoul, Dominic J. B. Hunter, Pascal Carrive, Emma Sierecki and Yann Gambin

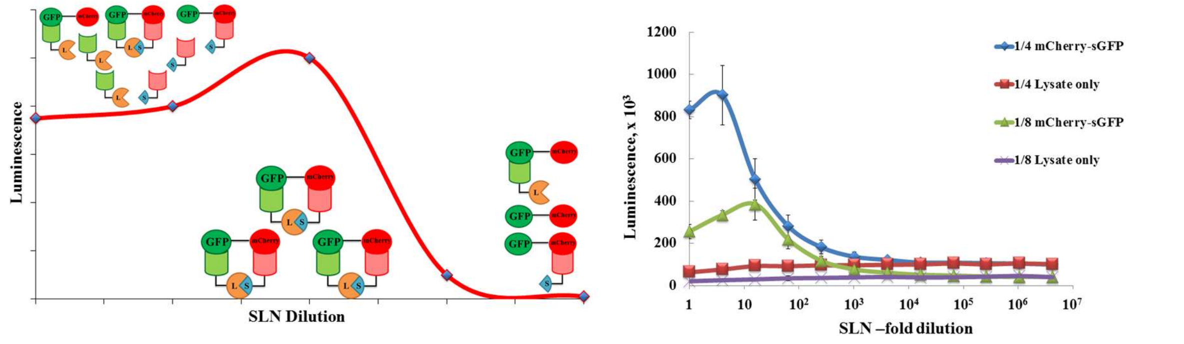

Figure S1 (Supplementary info): **A** – Typical 'hook' effect explained when the SLN is serially diluted instead of the analyte. At highest concentration of the SLN, the signal stays higher than background but doesn't reach maxima. Here, the SLNs are competing against each other for binding to their target where the two parts of split-Luc may end up on different protein targets. The signal reaches to maximum when the ratio between SLN and protein pair reaches 1:1 but drops down with further dilution due to lack of SLNs present for the reaction. **B** – Graphical representation of luminescence signal observed with serial dilution of SLN at a given concentration of analyte. Here, for each concentration analyte, we have corresponding concentration of non-expressing LTE that acts as background. It is evident that, at any given concentration of analyte, the signal is always higher than the background, confirming that we see limited split-Luc self-association and the complementation is driven by the presence of analyte.

Table S1: Protein sequences of the multiple protein used in construction of the universal split-Luc system.

Name of the Gene	Sequence
Anti – GFP	MQVQLVESGGALVQPGGSLRLSCAASGFPVNRYSMRWYRQAPGKEREWVAGMSSAGDRSSYEDSVKGRFTISRDDARNTVYLQMNSLKPEDTAVYYCNVNVGFEYWGQGTQVTVSS
Anti – mCherry	MAQVQLVESGGGLVQAGGSLRLSCATSGFTFSDYAMGWFRQAPGKEREFVAAISWSGHVTDYADSVKGRFTISRDNVKNTVYLQMNSLKPEDTAVYSCAAAKSGTWWYQRSENDFGSWGQGTQVTVSKEAI
Split-Luc Large bit	VFTLEDFVGDWEQTAAYNLDQVLEQGGVSSLLQNLAVSVTPIQRIVRSGENALKIDIHVIIPYEGLSADQMAQIEEVFKVVYPVDDHHFKVILPYGTLVIDGVTPNMLNYFGRPYEGIAVFDGKKITVTGTLWNGNKIIDERLITPDGSMLFRVTINS
Split-Luc Large bit	VTGYRLFEEIL
Flexible Linker	GSSGGGGSGGGGSSG
Rigid Linker	EAAAKEAAAK
Flexible-Rigid linker	GSSGGGGSGGGGSSGEAAAKEAAAKGSSGGGGSGGGGSSGEAAAKEAAAKGSSGGGGSGGGGSSG

Table S2: Accession numbers for the multiple proteins used as targets to test the universal split-Luc system

Name of Gene	Accession number
eGFP	DQ389577.1
sfGFP	ASL68970
mCherry	AKH87425
Foldon	4NCV_A
cMyc (b/HLH/Zip)	P01106
HDAC3	O15379
SOX9	P48436
MyD88	Q99836
Cav1	Q03135
MaxZ (b/HLH/Zip)	P61244
